# 2-(4-Nitro­phen­yl)-5-phenyl­furan

**DOI:** 10.1107/S160053680905288X

**Published:** 2009-12-12

**Authors:** Zhi Guan, Yan-hong He, Gangqiang Wang

**Affiliations:** a School of Chemistry and Chemical Engineering, Southwest University, Chongqing 400715, People’s Republic of China

## Abstract

The mol­ecular skeleton of the title mol­ecule, C_16_H_11_NO_3_, is nearly planar with the two aromatic rings forming a dihedral angle of 2.73 (7)°. In the crystal, weak inter­molecular C—H⋯O hydrogen bonds link mol­ecules into ribbons extended along [101]. The crystal packing exhibits also π–π inter­actions, as indicated by the short centroid–centroid distances between the aromatic rings [3.681 (3) Å] and between the aromatic and furan rings [3.811 (3) Å] of neighbouring mol­ecules.

## Related literature

For details of the synthesis, see: Wang *et al.* (2009 ▶).
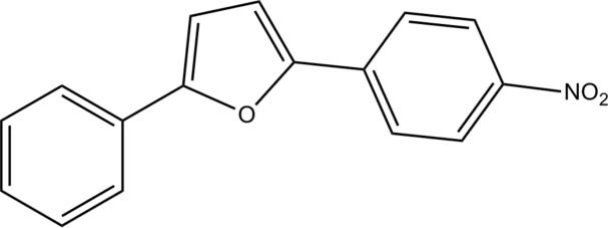


## Experimental

### 

#### Crystal data

C_16_H_11_NO_3_
*M* *_r_* = 265.26
Monoclinic, 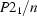

*a* = 7.3213 (15) Å
*b* = 16.290 (3) Å
*c* = 10.904 (2) Å
β = 100.81 (3)°
*V* = 1277.3 (4) Å^3^
*Z* = 4
Mo *K*α radiation
μ = 0.10 mm^−1^
*T* = 113 K
0.24 × 0.22 × 0.19 mm

#### Data collection

Rigaku Saturn CCD area-detector diffractometer
Absorption correction: multi-scan (*CrystalClear*; Rigaku/MSC, 2005 ▶) *T*_min_ = 0.977, *T*_max_ = 0.982
10136 measured reflections
2924 independent reflections
1168 reflections with *I* > 2σ(*I*)
*R*_int_ = 0.070

#### Refinement

*R*[*F* ^2^ > 2σ(*F* ^2^)] = 0.063
*wR*(*F* ^2^) = 0.174
*S* = 1.00
2924 reflections
182 parameters
H-atom parameters constrained
Δρ_max_ = 0.22 e Å^−3^
Δρ_min_ = −0.19 e Å^−3^

### 

Data collection: *CrystalClear* (Rigaku/MSC, 2005 ▶); cell refinement: *CrystalClear*; data reduction: *CrystalClear*; program(s) used to solve structure: *SHELXS97* (Sheldrick, 2008 ▶); program(s) used to refine structure: *SHELXL97* (Sheldrick, 2008 ▶); molecular graphics: *ORTEP-3* (Farrugia, 1997 ▶); software used to prepare material for publication: *SHELXL97*.

## Supplementary Material

Crystal structure: contains datablocks I, global. DOI: 10.1107/S160053680905288X/cv2666sup1.cif

Structure factors: contains datablocks I. DOI: 10.1107/S160053680905288X/cv2666Isup2.hkl

Additional supplementary materials:  crystallographic information; 3D view; checkCIF report

## Figures and Tables

**Table 1:** Hydrogen-bond geometry (Å, °)

*D*—H⋯*A*	*D*—H	H⋯*A*	*D*⋯*A*	*D*—H⋯*A*
C3—H3⋯O3^i^	0.95	2.51	3.373 (3)	152
C12—H12⋯O2^ii^	0.95	2.61	3.435 (3)	146

Symmetry codes: (i) 
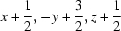
; (ii) 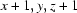.

**Fig. 1.**
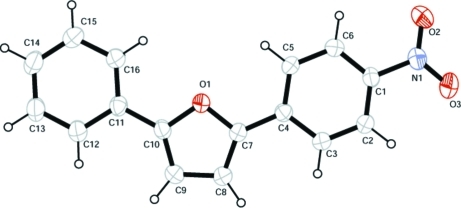
The molecular structure of the title molecule, with the atom-numbering scheme. Displacement ellipsoids are drawn at the 30% probability level.

## supplementary crystallographic information

### Comment

The title compound, (I), has been obtained as a by-product in our ongoing research of highly substituted furan derivatives (Wang *et al.*, 2009).

In (I) (Fig. 1), two aromatic rings form a dihedral angle of 2.73 (7) °. Weak intermolecular C—H···O hydrogen bonds (Table 1) and π-π stacking interactions with centroid-centroid separations of 3.681 (3) and 3.811 (3) Å consolidate the crystal packing.

### Experimental

A solution of ethyl 2-benzoyl-4-(4-nitrophenyl)-4-oxobutanoate (0.353 g, 0.7 mmol) in ionic liquid [bmim]HSO_4 _(1.6 g, 6.7 mmol) was stirred at 150 °C for 4 h in oil bath. After cooling to r.t., the reaction mixture was extracted with diethyl ether thoroughly. The combined extracts were washed with water, brine,dried (Na_2_SO_4_),and filtered. The solvents were removed and the residue was purified by flash chromatography (petroleum ether / dichloromethane 2:1) to get Ethyl 5-(4-nitrophenyl)-2-phenylfuran-3-carboxylate (240 mg, 71%) and the title compound (50 mg,14%) as yellow crystals.

### Refinement

All H-atoms were positioned geometrically and refined using a riding model, with d(C—H) = 0.95 Å, *U*_iso_=1.2*U*_eq_(C).

### Crystal data

**Table:**

C_16_H_11_NO_3_	*F*(000) = 552
*M**_r_* = 265.26	*D*_x_ = 1.379 Mg m^−^^3^
Monoclinic, *P*2_1_/*n*	Mo *K*α radiation, λ = 0.71073 Å
*a* = 7.3213 (15) Å	Cell parameters from 3226 reflections
*b* = 16.290 (3) Å	θ = 2.3–27.7°
*c* = 10.904 (2) Å	µ = 0.10 mm^−^^1^
β = 100.81 (3)°	*T* = 113 K
*V* = 1277.3 (4) Å^3^	Block, orange
*Z* = 4	0.24 × 0.22 × 0.19 mm

### Data collection

**Table:**

Rigaku Saturn CCD area-detector diffractometer	2924 independent reflections
Radiation source: rotating anode	1168 reflections with *I* > 2σ(*I*)
confocal	*R*_int_ = 0.070
Detector resolution: 7.31 pixels mm^-1^	θ_max_ = 27.6°, θ_min_ = 3.1°
ω and φ scans	*h* = −9→9
Absorption correction: multi-scan (*CrystalClear*; Rigaku/MSC, 2005)	*k* = −21→21
*T*_min_ = 0.977, *T*_max_ = 0.982	*l* = −12→14
10136 measured reflections	

### Refinement

**Table:**

Refinement on *F*^2^	Hydrogen site location: inferred from neighbouring sites
Least-squares matrix: full	H-atom parameters constrained
*R*[*F*^2^ > 2σ(*F*^2^)] = 0.063	*w* = 1/[σ^2^(*F*_o_^2^) + (0.0654*P*)^2^] where *P* = (*F*_o_^2^ + 2*F*_c_^2^)/3
*wR*(*F*^2^) = 0.174	(Δ/σ)_max_ < 0.001
*S* = 1.00	Δρ_max_ = 0.22 e Å^−^^3^
2924 reflections	Δρ_min_ = −0.19 e Å^−^^3^
182 parameters	Extinction correction: *SHELXL97* (Sheldrick, 2008), Fc^*^=kFc[1+0.001xFc^2^λ^3^/sin(2θ)]^-1/4^
Primary atom site location: structure-invariant direct methods	Extinction coefficient: 0.086 (10)
Secondary atom site location: difference Fourier map	

### Special details

**Table:**

Geometry. All e.s.d.'s (except the e.s.d. in the dihedral angle between two l.s. planes) are estimated using the full covariance matrix. The cell e.s.d.'s are taken into account individually in the estimation of e.s.d.'s in distances, angles and torsion angles; correlations between e.s.d.'s in cell parameters are only used when they are defined by crystal symmetry. An approximate (isotropic) treatment of cell e.s.d.'s is used for estimating e.s.d.'s involving l.s. planes.
Refinement. Refinement of *F*^2^ against ALL reflections. The weighted *R*-factor *wR* and goodness of fit *S* are based on *F*^2^, conventional *R*-factors *R* are based on *F*, with *F* set to zero for negative *F*^2^. The threshold expression of *F*^2^ > σ(*F*^2^) is used only for calculating *R*-factors(gt) *etc*. and is not relevant to the choice of reflections for refinement. *R*-factors based on *F*^2^ are statistically about twice as large as those based on *F*, and *R*- factors based on ALL data will be even larger.

### Fractional atomic coordinates and isotropic or equivalent isotropic displacement parameters (Å^2^)

**Table:**

	*x*	*y*	*z*	*U*_iso_*/*U*_eq_	
O1	0.83658 (18)	0.47058 (7)	0.09998 (13)	0.0540 (5)	
O2	0.3323 (3)	0.56325 (12)	−0.48419 (15)	0.0968 (7)	
O3	0.3467 (3)	0.69148 (10)	−0.43913 (17)	0.0971 (7)	
N1	0.3792 (3)	0.61960 (13)	−0.41169 (19)	0.0685 (6)	
C1	0.4782 (3)	0.60055 (13)	−0.2862 (2)	0.0528 (6)	
C2	0.5230 (3)	0.66262 (13)	−0.2022 (2)	0.0632 (7)	
H2	0.4856	0.7174	−0.2240	0.076*	
C3	0.6219 (3)	0.64521 (13)	−0.0866 (2)	0.0612 (7)	
H3	0.6524	0.6882	−0.0275	0.073*	
C4	0.6791 (3)	0.56563 (12)	−0.05378 (19)	0.0504 (6)	
C5	0.6301 (3)	0.50391 (12)	−0.1400 (2)	0.0569 (6)	
H5	0.6657	0.4489	−0.1185	0.068*	
C6	0.5298 (3)	0.52119 (12)	−0.2572 (2)	0.0595 (6)	
H6	0.4971	0.4787	−0.3169	0.071*	
C7	0.7901 (3)	0.55033 (12)	0.0684 (2)	0.0554 (6)	
C9	0.9599 (3)	0.54956 (13)	0.2595 (2)	0.0696 (8)	
H9	1.0268	0.5672	0.3382	0.084*	
C8	0.8639 (3)	0.59996 (14)	0.1642 (2)	0.0700 (8)	
H8	0.8530	0.6580	0.1666	0.084*	
C10	0.9392 (3)	0.47133 (13)	0.2184 (2)	0.0547 (6)	
C11	1.0059 (3)	0.39286 (13)	0.2725 (2)	0.0552 (6)	
C12	1.0985 (3)	0.38948 (14)	0.3963 (2)	0.0673 (7)	
H12	1.1158	0.4382	0.4450	0.081*	
C13	1.1648 (3)	0.31612 (15)	0.4481 (2)	0.0787 (8)	
H13	1.2278	0.3145	0.5326	0.094*	
C14	1.1415 (4)	0.24587 (16)	0.3802 (3)	0.0848 (9)	
H14	1.1872	0.1953	0.4173	0.102*	
C15	1.0523 (4)	0.24782 (14)	0.2581 (3)	0.0847 (9)	
H15	1.0371	0.1986	0.2104	0.102*	
C16	0.9841 (3)	0.32104 (13)	0.2038 (2)	0.0683 (7)	
H16	0.9221	0.3220	0.1191	0.082*	

### Atomic displacement parameters (Å^2^)

**Table:**

	*U*^11^	*U*^22^	*U*^33^	*U*^12^	*U*^13^	*U*^23^
O1	0.0608 (9)	0.0479 (9)	0.0501 (10)	0.0013 (7)	0.0021 (8)	0.0039 (7)
O2	0.1211 (16)	0.0947 (14)	0.0616 (12)	0.0146 (11)	−0.0160 (11)	−0.0143 (10)
O3	0.1200 (16)	0.0727 (13)	0.0855 (14)	0.0136 (10)	−0.0141 (12)	0.0234 (10)
N1	0.0684 (13)	0.0728 (14)	0.0594 (14)	0.0080 (10)	−0.0007 (11)	0.0052 (11)
C1	0.0501 (12)	0.0567 (13)	0.0489 (13)	0.0029 (10)	0.0023 (10)	0.0059 (10)
C2	0.0694 (15)	0.0474 (13)	0.0655 (16)	0.0056 (11)	−0.0063 (12)	0.0013 (11)
C3	0.0701 (15)	0.0471 (13)	0.0605 (15)	0.0021 (10)	−0.0031 (12)	−0.0038 (11)
C4	0.0537 (13)	0.0457 (12)	0.0507 (13)	−0.0042 (9)	0.0072 (10)	0.0037 (10)
C5	0.0726 (15)	0.0420 (12)	0.0540 (14)	0.0031 (10)	0.0063 (12)	0.0040 (10)
C6	0.0739 (16)	0.0502 (13)	0.0516 (14)	−0.0001 (10)	0.0048 (12)	−0.0039 (10)
C7	0.0614 (13)	0.0468 (12)	0.0547 (14)	0.0027 (10)	0.0029 (11)	0.0032 (10)
C9	0.0815 (17)	0.0579 (15)	0.0600 (15)	−0.0001 (12)	−0.0111 (13)	−0.0044 (12)
C8	0.0825 (17)	0.0520 (14)	0.0660 (16)	0.0046 (12)	−0.0102 (13)	−0.0033 (12)
C10	0.0535 (13)	0.0579 (14)	0.0484 (14)	−0.0008 (10)	−0.0015 (11)	0.0047 (10)
C11	0.0484 (12)	0.0554 (13)	0.0588 (15)	−0.0014 (10)	0.0023 (11)	0.0082 (11)
C12	0.0727 (16)	0.0644 (15)	0.0594 (16)	0.0006 (12)	−0.0015 (13)	0.0066 (12)
C13	0.0857 (18)	0.0737 (18)	0.0680 (18)	0.0021 (14)	−0.0076 (15)	0.0174 (14)
C14	0.100 (2)	0.0590 (16)	0.087 (2)	0.0009 (14)	−0.0049 (17)	0.0195 (14)
C15	0.106 (2)	0.0523 (15)	0.087 (2)	−0.0018 (13)	−0.0051 (17)	0.0046 (14)
C16	0.0772 (16)	0.0555 (14)	0.0641 (16)	−0.0037 (12)	−0.0074 (13)	0.0029 (12)

### Geometric parameters (Å, °)

**Table:**

O1—C10	1.367 (2)	C9—C10	1.350 (3)
O1—C7	1.371 (2)	C9—C8	1.405 (3)
O2—N1	1.218 (2)	C9—H9	0.9500
O3—N1	1.221 (2)	C8—H8	0.9500
N1—C1	1.457 (3)	C10—C11	1.453 (3)
C1—C2	1.363 (3)	C11—C16	1.382 (3)
C1—C6	1.367 (3)	C11—C12	1.394 (3)
C2—C3	1.360 (3)	C12—C13	1.371 (3)
C2—H2	0.9500	C12—H12	0.9500
C3—C4	1.388 (3)	C13—C14	1.357 (3)
C3—H3	0.9500	C13—H13	0.9500
C4—C5	1.377 (3)	C14—C15	1.369 (3)
C4—C7	1.446 (3)	C14—H14	0.9500
C5—C6	1.379 (3)	C15—C16	1.382 (3)
C5—H5	0.9500	C15—H15	0.9500
C6—H6	0.9500	C16—H16	0.9500
C7—C8	1.351 (3)		
			
C10—O1—C7	107.20 (15)	C10—C9—H9	126.4
O2—N1—O3	123.0 (2)	C8—C9—H9	126.4
O2—N1—C1	118.64 (19)	C7—C8—C9	107.1 (2)
O3—N1—C1	118.31 (19)	C7—C8—H8	126.4
C2—C1—C6	121.7 (2)	C9—C8—H8	126.4
C2—C1—N1	119.23 (19)	C9—C10—O1	109.24 (17)
C6—C1—N1	119.06 (19)	C9—C10—C11	133.4 (2)
C3—C2—C1	119.2 (2)	O1—C10—C11	117.31 (18)
C3—C2—H2	120.4	C16—C11—C12	118.48 (19)
C1—C2—H2	120.4	C16—C11—C10	122.0 (2)
C2—C3—C4	121.1 (2)	C12—C11—C10	119.55 (19)
C2—C3—H3	119.5	C13—C12—C11	120.3 (2)
C4—C3—H3	119.5	C13—C12—H12	119.9
C5—C4—C3	118.56 (19)	C11—C12—H12	119.9
C5—C4—C7	122.16 (18)	C14—C13—C12	120.8 (2)
C3—C4—C7	119.27 (18)	C14—C13—H13	119.6
C4—C5—C6	120.60 (19)	C12—C13—H13	119.6
C4—C5—H5	119.7	C13—C14—C15	120.0 (2)
C6—C5—H5	119.7	C13—C14—H14	120.0
C1—C6—C5	118.91 (19)	C15—C14—H14	120.0
C1—C6—H6	120.5	C14—C15—C16	120.3 (2)
C5—C6—H6	120.5	C14—C15—H15	119.9
C8—C7—O1	109.16 (18)	C16—C15—H15	119.9
C8—C7—C4	133.11 (19)	C11—C16—C15	120.2 (2)
O1—C7—C4	117.73 (17)	C11—C16—H16	119.9
C10—C9—C8	107.3 (2)	C15—C16—H16	119.9
			
O2—N1—C1—C2	−176.6 (2)	O1—C7—C8—C9	−0.1 (3)
O3—N1—C1—C2	3.1 (3)	C4—C7—C8—C9	−179.7 (2)
O2—N1—C1—C6	5.8 (3)	C10—C9—C8—C7	−0.6 (3)
O3—N1—C1—C6	−174.4 (2)	C8—C9—C10—O1	1.0 (3)
C6—C1—C2—C3	0.2 (4)	C8—C9—C10—C11	179.3 (2)
N1—C1—C2—C3	−177.37 (19)	C7—O1—C10—C9	−1.1 (2)
C1—C2—C3—C4	0.6 (4)	C7—O1—C10—C11	−179.68 (19)
C2—C3—C4—C5	−1.4 (3)	C9—C10—C11—C16	−171.6 (2)
C2—C3—C4—C7	177.7 (2)	O1—C10—C11—C16	6.5 (3)
C3—C4—C5—C6	1.4 (3)	C9—C10—C11—C12	6.9 (4)
C7—C4—C5—C6	−177.6 (2)	O1—C10—C11—C12	−174.98 (19)
C2—C1—C6—C5	−0.2 (4)	C16—C11—C12—C13	−0.4 (4)
N1—C1—C6—C5	177.36 (19)	C10—C11—C12—C13	−179.0 (2)
C4—C5—C6—C1	−0.6 (4)	C11—C12—C13—C14	0.0 (4)
C10—O1—C7—C8	0.8 (2)	C12—C13—C14—C15	0.5 (4)
C10—O1—C7—C4	−179.61 (18)	C13—C14—C15—C16	−0.6 (4)
C5—C4—C7—C8	175.4 (2)	C12—C11—C16—C15	0.4 (4)
C3—C4—C7—C8	−3.6 (4)	C10—C11—C16—C15	178.9 (2)
C5—C4—C7—O1	−4.1 (3)	C14—C15—C16—C11	0.1 (4)
C3—C4—C7—O1	176.93 (19)		

### Hydrogen-bond geometry (Å, °)

**Table:**

*D*—H···*A*	*D*—H	H···*A*	*D*···*A*	*D*—H···*A*
C3—H3···O3^i^	0.95	2.51	3.373 (3)	152
C12—H12···O2^ii^	0.95	2.61	3.435 (3)	146

Symmetry codes: (i) *x*+1/2, −*y*+3/2, *z*+1/2; (ii) *x*+1, *y*, *z*+1.
